# Accelerating High-Throughput Screening for Structural Materials with Production Management Methods

**DOI:** 10.3390/ma11081330

**Published:** 2018-08-01

**Authors:** Alexander Bader, Finn Meiners, Kirsten Tracht

**Affiliations:** Bremen Institute of Mechanical Engineering (BIME), University of Bremen, Badgasteiner Str. 1, 28359 Bremen, Germany; meiners@bime.de (F.M.); tracht@bime.de (K.T.)

**Keywords:** micro-manufacturing, manufacturing systems, production planning

## Abstract

High-throughput screenings are widely accepted for pharmaceutical developments for new substances and the development of new drugs with required characteristics by evolutionary studies. Current research projects transfer this principle of high-throughput testing to the development of metallic materials. In addition to new generating and testing methods, these types of high-throughput systems need a logistical control and handling method to reduce throughput time to get test results faster. Instead of the direct material flow found in classical high-throughput screenings, these systems have a very complex structure of material flow. The result is a highly dynamic system that includes short-term changes such as rerun stations, partial tests, and temporarily paced sequences between working systems. This paper presents a framework that divides the actions for system acceleration into three main sections. First, methods for special applications in high-throughput systems are designed or adapted to speed up the generation, treatment, and testing processes. Second, methods are needed to process trial plans and to control test orders, which can efficiently reduce waiting times. The third part of the framework describes procedures for handling samples. This reduces non-productive times and reduces order processing in individual lots.

## 1. Introduction

**Figure 1:**
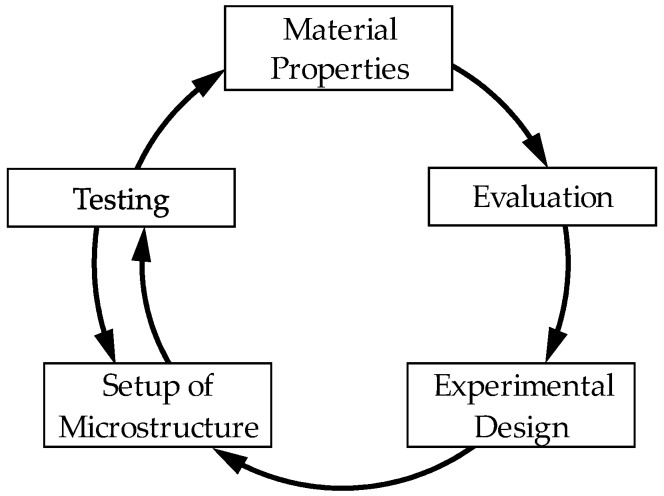
Schematic procedure of the method ‘Farbige Zustände’ [4].

Steels and aluminum alloys can be used as structural materials, however, they may not have satisfactory material properties for a given use. For these areas of application, adapted materials must be developed so that components can fulfil their intended structural function. The mechanical properties result from interactions between alloy composition and microstructure, which must be coordinated in many investigation steps. In conventional research and development, the product is adapted to the material properties [1]. Therefore, it is necessary to define alloy composition and the setup of microstructure to meet required performance profiles. In this context, Springer and Raabe have developed a combinatorial process with which varying alloy compositions and heat treatments are systematically investigated by means of tensile tests [2]. Research into these new compositions of alloy and microstructure with conventional, standardized methods of materials testing requires a great amount of time and material effort. For speeding up the testing of materials and identification, Ellendt and Mädler introduced a new approach for the development of structural materials that is based on the identification of materials in high-throughput. This high-throughput system can perform examinations in a few hours or days that would take weeks or months using conventional methods. In order to increase the productivity of high-throughput screening and further shorten the development time, the targeted use of screening strategies is necessary [3]. Within the evolutionary high-throughput system ‘Farbige Zustände’, metallic materials are developed [4]. This system enables the identification of new alloys, which meet specific performance profiles, defined by mechanical material properties, such as the hardness, the elongation at fracture, the young’s module, and other factors. Through statistical and heuristical processes, an experimental plan is calculated, which shows all steps for the production and setup of the alloy’s microstructure as well as for the examination to determine the characteristics of the alloy (Figure 1). To achieve these properties, in addition to the alloy composition, the targeted thermal and mechanical treatment of the samples is defined [5].

The high-throughput system ‘Farbige Zustände’ greatly differs from conventional screenings regarding its structure. Conventional screenings of pharmaceutical products or catalyst materials usually have a linear material flow because these samples are not able to go through a processing station more than once. High-throughput screening consists of interlinked individual stations, each of which carries out investigations on samples. These tests are fully automated and optimized for high throughput. On this basis, high-throughput systems became established in the late 1990s [6]. However, no intelligent experiments are carried out or combinatorial strategies are applied [7]. In this system, adapted methods reduce examinations, which usually last weeks or months, to one or two hours with the same application rate [8]. This is possible because components, temperature, pressure, and other parameters in the high-throughput experiments can be changed. Especially important for successful implementation of high-throughput systems are fast data processing and automated systems, which enable fast sample processing. This makes it possible to process up to 100,000 samples per day for so-called ultra-high-throughput analyses [9]. Research and development is being industrialized to further increase output and reduce development costs in the development of active ingredients. Industrialization is characterized in particular by the use of high-throughput systems. By means of automation, parallelization, and minimization of processes, these systems enable a faster acquisition of as much data as possible from experiments with the lowest possible material consumption [10]. Within high-throughput screenings for mechanical material testing, an individual composition of test plans for each target of analysis is required. Therefore, the high-throughput screening for structural materials has a high resemblance with job shop production. In such production systems, machines and working systems are arranged according to their functions and not according to the production order of one single product. Orders reach machines in a previously planned sequence for the completion of a component or assembly [11]. Due to the flexible material flow and the complexity of the system, which is also caused by the handling of micro samples, the material flow and the processing of the samples must be controlled. For this purpose, approaches from production technology are examined that lead to a reduction in throughput time in production systems. The focus is on the evaluation of individual processes as well as the entire system. The aim is to significantly increase throughput in order to be able to compare the performance of the high-throughput system with conventional high-throughput screening and to reduce nonproductive times by handling of parts.

## 2. Enhancement of Throughput Time in Production Systems

In recent years, the production of customized products and the associated flexibility of order processing have come into focus for production systems [12]. In this context, it is often referred to as one-offs [13]. Flexibility and reaction to short-term changes can be achieved primarily by reducing order throughput times [14]. According to Nyhuis and Wiendahl, there is a conflict of objectives with the throughput time and performance. Both show a clear change with the system’s inventory. Increasing inventory leads to both increased performance and longer throughput times, so a system-specific optimum operating point must be determined [15]. The main components of the throughput time are queuing times, transport times, and setup times. In this context, the proportion of processing time is significantly lower [16]. Nevertheless, the optimization of production systems often focuses on reducing the processing time. In general, processing times can be planned and offer a reliable savings potential. Transport times, but above all queuing times, are characterized by a large stochastic proportion of time, which makes it difficult or even impossible to plan these time-shares. However, it was shown by Nyhuis and Wiendahl that reduction of the stock has an especially strong positive effect on the resting time [15].

**Figure 2:**
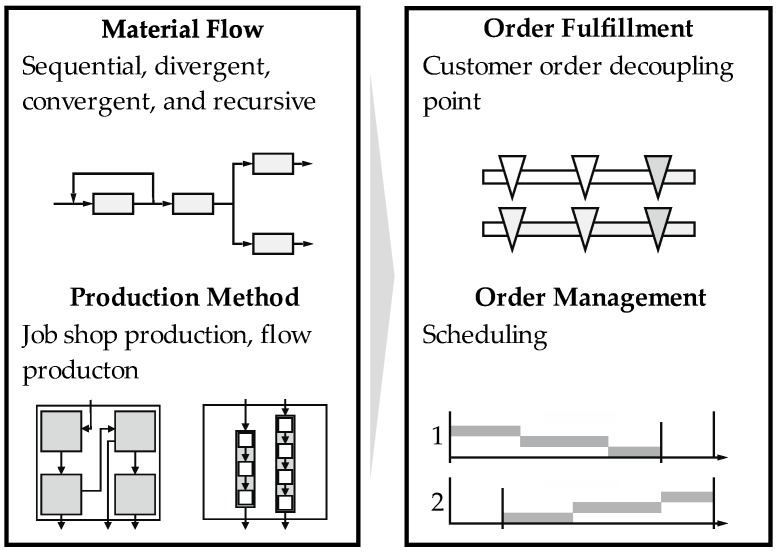
Tasks of production management.

Production systems are factories or production lines in which a value-adding activity is carried out. At the end of these activities, usually finished components or assemblies and products are produced. These systems are supported by methods and strategies to ensure the optimal organization of the system. The methods describe an integrated production organization that consists of effective and efficient collaborative subsystems, work systems, concepts, methods, and tools. The focus is on customer benefit and should be achieved while avoiding waste [17]. For the successful organization of production systems, their characteristics and interaction with the customer are crucial. There are different types of orders for production systems, such as make-to-order or make-to-stock, and they are supplemented by mixed forms (see Figure 2). This shifts the customer decoupling point depending on the order type. According to the order type, different types of production can be selected, from shop floor production to paced continuous flow production. The order type determines the basic structure of production and the arrangement of machines and working systems. Therefore, they have an influence on the material flow and process type as well as on the entire order processing. In this context, orders involving a large number of a serial products must be organized in a different way in production than the manufacturing one-offs. This concerns both the arrangement and connection of machines, and the scheduling of production orders.

**Figure 3:**
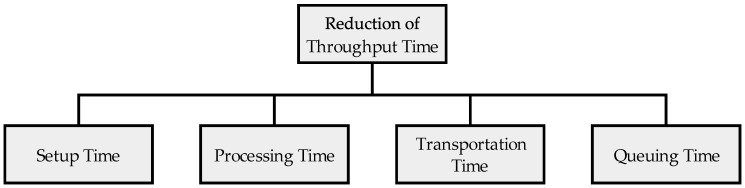
Reduction of manufacturing throughput time per part [18].

Johnson has designed a framework for reducing throughput time in production systems and described various methods that lead to a reduction in throughput time. Figure 3 shows Johnson’s classification of component changes that will reduce the Manufacturing Throughput Time per Part (MTTP). These changes are reduction of setup time, processing time, transportation time, and queuing time [18]. To reduce setup times, entire production lines are sometimes converted to enable faster set-ups. The use of standardized interfaces or a standardization of work piece carriers are conceivable options. In the automotive industry, entire product classes are combined on a modular platform, which also avoids the set-up of machines or the build-up of several parallel production lines. A typical procedure to fast equipment setups is the Single Minute Exchange of Die (SMED) method [19]. This reduces batch sizes and increases productivity [20,21].

Previous research describes various approaches to reduce process times in production. For example, other fast technologies can be used for production or assembly times can be reduced by integrating functions. A learning process for the workers can also lead to an improvement in process time. In addition to reducing machining time through other machine parameters, the number of operations can also be reduced by revising components and processes [18]. In 1994 Suresh and Meredith described in this context an increase in productivity that reduces throughput times by forming product families that are processed together in production [22]. The transportation time can be reduced by accelerating transport systems or reducing distance [18]. Likewise, a systematic determination of lot sizes can influence the transport time per part.

The queuing time makes up a particularly large proportion of the throughput time. Wiendahl has already found that queuing time represents the largest share of lead time at 75%, but processing time is less than 10% [16]. In production planning and control, the focus is therefore on reducing these waiting and idle times before processing. In this context, the dilemma of production was already formulated in the 1960s. Gutenberg describes this as a contradiction between maximizing utilization and minimizing throughput times [23]. Thus, it is not possible to achieve short throughput times and high performance together in one system. An explanation model for this connection is the funnel model. The stock of a working system can be represented as a funnel whose lower opening represents the current performance [11]. This means that an inventory in the funnel is required as a buffer so that capacity utilization can be ensured across performance fluctuations. Too much inventory fills the funnel and increases throughput times. However, if the inventory is too low, the maximum performance cannot be reached [24]. This correlation becomes clear in Nyhuis’ and Wiendahl’s characteristic curve theory. A direct connection between throughput time, performance, and inventory was established and mathematically documented for the first time. It became clear that the throughput time increases with growing inventory. At the same time, performance reaches its maximum value with increasing inventory [15].

The funnel model forms the framework for many methods of loading planning and production control in job shop production. Logistical control methods achieve short throughput times and high schedule reliability, resulting in higher flexibility and short delivery times [25]. The result is a customer-oriented system that needs to be capable of implementing one-offs. Production control is essential for a flexible and fast production, which is characterized by a high proportion of flowing material [26]. The aim of these methods is to set the inventory as the control parameter and use it to achieve logistical target criteria such as throughput time, utilization, and schedule reliability. The inventory is the total work that is in the system. Depending on the production systems and the company objectives, logistical target criteria are weighted, so that the method used for loading planning varies for each production system. For example, with the very simple Kanban principle, a shorter throughput time can be achieved than that of a control system using Constant Work-In-Process (CONWIP) [27]. Workload Control (WLC), on the other hand, has a positive influence on the schedule reliability and performance of a production system [28]. In the procedure by Bechte, called Load Oriented Order Release (LOOR), a stock list is maintained that lists both the direct stock of a work system and the indirect stock. The indirect stock includes work orders that reach the relevant work system in the future and are included with a discount factor. An order is only released in this method if the direct and indirect stock does not exceed a previously defined limit value [29]. Even today, many order release procedures are still based on the concept of controlling inventories.

Another approach to reduce waiting and queuing times is lean production. This method was developed in the 1980s at Toyota in Japan and has become known worldwide as the Toyota Production System (TPS) [30]. The aim is to reduce non-value-adding activities and minimize waste. The focus is exclusively on value-adding work [31]. Lean production also focuses on reducing inventories and waste as these are seen as a great potential for hiding inefficient processes. In this way, resources can be better used. A further development of lean production was further expanded to support the volatile and competitive business environment [32,33]. Continuous Improvement (CI), the introduction of pull principles and a zero-defect strategy in production by simplifying processes, are of particular importance in lean production. Employee orientation and goal-oriented leadership through visual management are also tools of lean management [34]. Thus, a Lean Production System (LPS) is created that provides for a company-specific orientation of the production system using the eight basic principles [35].

## 3. Acceleration of High-Throughput Systems

**Figure 4:**
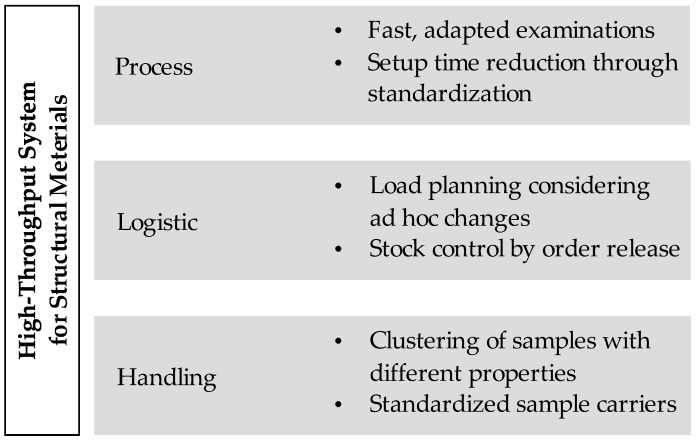
Framework for high-throughput system acceleration.

A classification between the characteristics of production systems and high-throughput systems shows comparable theories in organization, order management, material flow, order type, manufacturing type, and process organization. Similar to production organization, orders in high-throughput systems are controlled by a process chain, and processed at various workstations. Orders initiate activities in these systems. One kind of activity by order release is the definition of a fixed sequence. In addition, due dates and the number of samples are also specified while releasing the order [36]. Due to a similar behavior between production systems and high-throughput systems, many approaches from the production organization can be transferred in their principles to high-throughput systems (see Figure 4). In addition to the development of methods for process improvement, enhancements in transportation between stations and control as well as in handling contribute to a reduction in throughput time.

### 3.1. Development of Testing Processes

The ‘Farbige Zustände’ method requires the development of processes for short-term characterization that are suitable to describe structural materials in terms of their suitability for a given performance profile. In their approach to develop this high-throughput method, Ellendt and Mädler describe that previous, partially standardized methods for sample generation and material testing can be applied to a limited extent only, since the material and time requirements exceed the possibilities of the high-throughput system. Therefore, methods for the generation of metallic samples as well as for the treatment and testing of samples are developed or adapted for the high-throughput system [1]. This process is capable of producing several thousand individual samples per day. These must then be subjected to rapid processes to change and characterize the properties of the sample. These methods together form the basis for multi-stage screening. Micro samples are produced in a droplet generator for microscopic spheres, in which the sample size can be specifically adjusted by a controlled solidification process [37]. Thus, the investigation of new alloy compositions is based on the use of micro samples with a diameter of less than one millimeter. This combination saves both time in alloy evaluation and the amount of material [38]. The particular challenge is to determine the micro-properties, although the macro-properties differ significantly from the intended areas of application. The use of micro-samples results in special size effects that affect both the microstructure [39,40] and the macrostructure [40]. These are taken into account by a predictor-based optimization approach in the determination of micro process parameters, so that a high quality of information can be expected [5]. In this context, the use of microspheres offers the advantage that they exhibit homogeneous microstructure without macro segregations immediately after generation [4]. This structure is specifically adjusted in a subsequent heat treatment, which takes the scalability into account. Laser-deep-alloyed samples are also available. This process offers the generation of different alloy compositions and graded compositions in short intervals [41].

In addition to the alloy composition, the microstructure of materials is decisive for the mechanical properties of structural materials. These can be specifically modified by thermal and mechanical treatments. An example of this is the multistage contact deep rolling that produces an increase in surface integrity. For example, the rolling process can cause partial strain hardening of material and thus specifically adapt the mechanical properties of the sample [42]. These modified properties are investigated in various short-term characterization processes that allow conclusions to be made on the material properties of the samples [38]. In addition to methods of nanoindentation, X-ray diffraction and hardness measurement based on laser induced shock waves, a method for dilatometry, are part of the characterization process [4,43,44]. High-throughput dilatometry can be used to examine micro samples that have a high cooling rate due to their small volume and large surface area. Cramer et al. have developed a special dilatometer tailored to the needs of the high-throughput system, with which the thermal expansion of samples can be investigated [45]. These methods, which are developed with the aim of short process times, standardized sample transfer, and a specific testing target for high-throughput systems, form the basis for the development of a high-throughput system for materials testing.

### 3.2. Efficient Logistical Control for High-Throughput Systems

Logistics control already plays a central role in production today and essentially involves the coordination of industrial production. Acceleration of high-throughput systems cannot be achieved solely through technological improvements. Due to the complexity of the system, the enhancement of the logistical targets has to be expanded by organizational methods. Procedures for logistical control for high-throughput testing in materials testing are developed on the model of production control. The findings of the production are applied to high-throughput systems and adapted to their requirements. This way, a high throughput and a short throughput time of test orders will be achieved. For the use in high-throughput systems, the order generation, order release, sequencing, and capacity control are of great importance, these assume the central tasks for order organization and have a high influence on the logistical targets. The aim is to achieve an optimum of short throughput times, high throughput, and high utilization when planning the schedule of high-throughput tests.

The objective for the use of planning and control methods in high-throughput systems for materials testing is to improve reactivity, flexibility, and throughput. This sometimes results in boundary conditions that are untypical for other high-throughput systems. Schneider et al. describe that stations in conventional material testing are decoupled and not connected by an automatic logistics unit. Instead, samples are often transported manually between the test stations [36]. This results in an undirected material flow, which is also present in a variable process sequence. An individual test plan is created for each sample, which only contains tests that are assigned to a specific test objective. These can also be influenced by the sample characteristics and the test results already available. Furthermore, it may be necessary for material tests that stations have to work within temporarily paced sequences. This results in a variable pace in these sections, where orders have to be processed in certain time slots [46]. It is also necessary to provide decision patterns for re-tests and partial tests in the logistical control in which a test is carried out a second time or carried out partially [47]. Thus, special requirements for logistical controls for high-throughput systems mainly comprise the material flow complexity, the number of variants, and the production principle (see Table 1). In addition, Nyhuis and Wiendahl described criteria to be observed for the implementation of a production control system [15]. These criteria must also be taken into account when selecting a suitable control system due to the similar structure and organization of high-throughput and production systems. In particular, a distinction between make-to-order and make-to-stock or job shop and flow production is fundamental. In addition, the qualification and motivation of employees must also be taken into account as so-called soft factors in the development of management [15].

**Figure 5:**
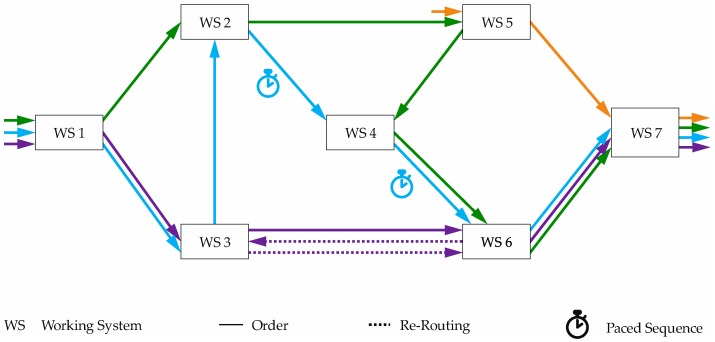
Structure of high-throughput systems for material testing [46].

The control method for high-throughput systems is capable of minimizing the effects of ad hoc changes, sample re-routing, temporary paced sequences, and quantity changes in order throughput. Thus, the throughput and flexibility of the system are to be maintained. In the case of ad hoc changes, the test plan of orders is changed spontaneously so that these samples follow a different process chain through the system. This can occur, for example, due to an overload of the originally planned work systems or by changing the test plan on the basis of measured values. Figure 5 shows process chains of different orders in an exemplary system. The blue, green, purple, and yellow jobs have a different order through the system so that they represent ad hoc changes, rerouting of samples, and temporary concatenation. The purple order is re-routed on the basis of a repeat check. The order is forwarded to working system (WS) 6 after being processed at WS 3. Then, the step at WS 3 and the test at WS 6 are repeated. The blue order represents the throughput of a paced order. It is assumed that the machining operations on working systems 2, 4, and 6 must take place in a paced sequence. Therefore, these working systems are treated as virtual flow production and the order is controlled accordingly. As an example of ad hoc changes, the orange order controls the working systems 5 and 7 at short notice, while the green order explains the effects of changes in unit quantities.

In addition to the tasks of short-term control, logistical planning and control must also include medium-term planning for a machine schedule. Schedule planning tasks include scheduling orders and machines. This results in detailed scheduling taking the current capacity into account. Based on the order and system data, forward scheduling is carried out for each order, without considering the order’s situation. The earliest possible start and end date is determined. Conventional throughput time scheduling has been supplemented by additional block times to take dynamic paced sequences into account. As soon as a station is processed in a paced sequence, all other stations of this sequence must be reserved for a seamless connection. In addition, the orders are sorted according to urgent and normal orders to ensure fast processing of higher-priority orders in overloaded work systems. Normal orders are sorted according to the First-in-System-First-out (FiSFo) rule to generate even throughput times. In subsequent capacity requirements planning, capacity buffers must be planned in order to be able to react to unforeseeable changes in the demand program. The capacity requirements planning can be used to identify overloads [48].

Based on this planning status, the orders are released for processing and checking in the short-term logistical control system. To do so, a high-throughput-specific sequence of orders is first created. A typical procedure for this is the use of the planned due date from the schedule. The closer the planned due date is to the current time, the further forward the order is taken into account. The structured orders can then be checked for order release. A special challenge for order release has turned out to be the consideration of repeat and partial tests, as well as the efficient control of temporarily timed sequences. Onken et al. presented an approach, in which a reduction in throughput time and the proportion of blockages in the system was achieved through specific order release. The use of a modified Decentralized Work in Process-oriented Manufacturing Control (DEWIP) also significantly increased the output of individual work systems [47]. A further development of the logistic control regarding the consideration of temporarily paced sequences also shows a further reduction of the throughput time compared to order release using the FiSFo. The investigations have shown that even with the use of an order release regulation, the throughput time is reduced by more than 50 percent [47]. A machine-specific analysis of capacity utilization has shown that it fluctuates widely and is in some cases below ten percent. For other work systems, good capacity utilization of over 90 percent is achieved [46]. Schedule planning has not been taken into account, which can be expected to improve the utilization significantly.

### 3.3. Multimodal Handling

The beginning of technologies for enabling high-throughput can be marked with the invention of the micro titer plate in the 1950s, a carrier for a multitude of liquid samples that fast became a standard in the field. In the following decades, the efficiency was increased by increasing the automation, with automated plate readers and multichannel pipettes [49]. The size of the sample was more and more reduced by introducing 96-, 384-, and 1536-well plates, which reduce the cost, time, and amount of material. While a 96-well plate carried 100–200 µL, the sample size on a 384-well plate was reduced to typically around 50 µL and to 5 µL on a 1536-well plate. Even 3456-well plates have been reported, but these small samples have not been established in the field [50].

The example of HTS in chemistry and biology show the requirements and catalyzers for high-throughput systems, which are easy handling on standardized carriers, automation, parallelization, and miniaturization. These basic concepts of high-throughput systems have also been applied in other fields, as for example materials research. Examples include the development of thermoelectric materials [51] and the development of abrasion resistive surfaces [52]. In these cases, the objects under test are functional materials, which can be tested by their surface characteristics. This allows for easy sample creation by using surface technologies like physical vapor deposition (PVD). The samples are created directly on a substrate, which acts as a sample carrier. This means that many examinations can be carried out on such large-area samples.

For the development of structural materials, most tests cannot be performed on thin films on a carrier. Tests like nanoindentation or hardness measurement based on laser-induced shock waves require a sample as bulk material, due to changes made to the geometry and microstructure of the processes. To provide these, the generated microspheres are used as samples. Similar to the process in chemistry, these samples can be created in high masses with gradually changing characteristics. The most important difference to previous high-throughput systems is the existence of single samples, which have to be prepared individually for the different testing stations within the high-throughput system. Due to the size of the spheres and the required low handling times, an individual handling of the samples raises new challenges for the automation. In order to achieve a system with high throughput, methods from production engineering have to be applied. A particular challenge is that the adhesion forces in the micro area can exceed the weight force. Adhesion forces are Van der Waals forces, capillary forces, and electrostatic forces [53]. Two different concepts are known for the handling of micro components. Similar to macro and meso components, micro components can be handled individually. They can be handled by grasping and moving them with the help of micro grippers, or without grasping them with contactless methods, for example with electrostatic or electromagnetic forces [54]. Fantoni et al. lists magnetic, suction, and Van-der-Waals grippers in addition to conventional frictions grippers [55], which use the size effects of micro components in a targeted manner [56]. This makes it difficult to release and place the components, for which active and passive systems can be used. The challenge is precise and controlled depositing, which can, however, be compensated with additional technology such as measuring systems or targeted introduction of force into the component [55,57].

If micro parts are made from semi-finished products, the application of linked parts can be reasonable. During production, micro-components are left in the semi-finished product that is used as conveying and positioning equipment. It enables an easier handling of the parts in flow productions. As long as the parts are linked together, they can be handled as macro material. Depending on the connection between the individual parts, the linkage can be described as line-compound, ladder-compound, or comb-compound [58]. In the case of a totally linear processing, the linked parts could run through the whole system without any costly reordering. In the case of a multi-dimensional factorial experiment, the effort for separating and reconnecting the linked parts would grow fast. In this case, the system cannot benefit from its strengths.

In order to optimize the throughput of the system, it has to be possible to create optimized batches for different processes. Therefore, a core requirement is the possibility of relocating individual samples and grouping them together to benefit from a parallelized handling in batches. Because of the multitude of different generating, treatment, and testing processes with different requirements, a multimodal handling system is used, which can transfer between different preparation methods and relocate samples for optimized batch processing. Examples for different modes are bulk handling, separation in trays, and fixated samples on a carrier. For the fixated handling on carriers, there are different types, depending on the requirements of the actual process. These may be requirements to the resistance to vertical and lateral forces, temperatures, chemicals, or other requirements as for example electrical conductivity. A constant identification of the components is ensured in order for the sphere’s position to be linked to the obtained measurement results.

In order to allow relocation, any fixation method must be releasable. A promising method for the fixation of microspheres, which allows fast fixation and release with a small temperature impact, is the embedding in low melting alloys. These are alloys, which have melting points as low as room temperature [59]. This ensures damage-free dissolution of the samples, without changing their mechanical properties and microstructure in a high-speed handling, in order to prevent bottlenecks caused by embedding and re-bedding. Thus, complex process chains with different sample preparation requirements are achieved for each treatment or testing process.

## 4. Discussion

The acceleration of high-throughput systems for testing structural materials will be achieved through a holistic approach. Aspects of production, setup of microstructure, and test processes as well as aspects of logistics and handling must be considered. Actions must be taken in each of the three areas to accelerate these areas individually. This is the only way to introduce high-throughput screening for testing structural materials. The validation of fast methods for material characterization is particularly important for implantation, since conventional material testing methods cannot be performed on microspheres. However, due to their rapid generation in the droplet generator and their homogeneous microstructure, these are well suited as samples within high-throughput screening.

The acceleration of the entire high-throughput screening process by adapting logistical concepts from production management is promising. Simulations have shown success in reducing throughput times and increasing capacity utilization by using individual components of logistical control. However, further research needs to be addressed in this context, as the improvement achieved does not yet allow for highly productive operation. Further methods and targets need to be developed that reflect the order requirements of individual work systems more clearly. Overall, a fully developed high-throughput system for testing structural materials can thus be used in all areas of design and development. Especially in combination with the development of highly stressed components or the need for special component properties, material development with the ‘Farbige Zustände’ method is useful.

The completed high-throughput system will give new insights into the principle relations between microstructure and mechanical properties of the material. On the one hand, the predictor function itself will point out connections between measured characteristics and microstructure. These connections may help the scientists to better understand the basic principles of the material’s behavior. On the other hand, the high-throughput system ‘Farbige Zustände’ as a tool can not only be used to find purpose specific materials, but also to get a better overview of the possibilities of material and structure combinations. The fast process sheds light on the white spots in the homogenous parameter space of material composition and treatment. The high-throughput system will allow researchers to find interesting compositions and to investigate them faster than ever before. For the industry, the fully developed high-throughput system will enable the use of specifically fitted materials for their requirements. This results, for example, in lighter or stringer parts, which will help to save energy or make products more durable.

## 5. Summary and Outlook

High-throughput systems for testing structural materials offer an approach to rapid characterization of material properties. The use of micro-samples allows for the investigation of a large number of alloy compositions with a small material and time input. In addition, various mechanical and thermal treatment processes are available to change the microstructure and thus the material properties. A fast generation of defined metallic samples in combination with fast tests enables the combination to a high-throughput screening. In addition, initial studies show that a logistical optimization can reduce throughput times by more than 50 percent. In contrast to conventional high-throughput screenings, systems for the examination of structural materials show a dynamic, non-directional material flow. Overall, the complexity of the system is increasing, so that actions must be taken to organize the system. This is a well-known problem in the field of production planning and organization. In industrial production systems, the focus is usually on economical operation and high added value. In this context, many processes have been developed that enable a productive and flexible production system. Transferring these approaches to high-throughput systems enables an increase in performance and a reduction in throughput time.

The framework for the acceleration of high-throughput systems makes partial use of these methods. A reduction of the throughput time in high-throughput systems can be achieved through the areas of process, logistics, and handling acceleration. The improvement of the individual processes in terms of throughput time is achieved by developing methods that are adapted to the aim of the analysis and the used samples. In this context, standardized sample carriers are used, which have uniform interfaces and are used for various types of examinations. This also provides an advantage when handling the samples to be tested. A standardized interface enables fast onward transport without any re-embedding processes in the meantime. In addition, these sample carriers offer the possibility of combining samples of different properties in one batch and reducing handling effort. As a third component, the framework for the acceleration of high-throughput systems includes measures for the efficient organization of the system. The focus is on load planning, taking ad hoc changes like re-tests, partial testing, and temporarily paced sequences into account. Inventory control through the use of a logistical control system is also part of order processing.

The development of the high-throughput system has mainly been carried out on individual components. Especially for the logistic approaches, an evaluation of the overall system is important. In this context, future work will create an organizational connection between the individual processes in order to induce interactions between work systems and logistical control. It is to be expected that order combinations will be generated by the test planning and logistic control, which will achieve synergy for better system utilization. In this way, the system’s productivity will be increased overall.

## Figures and Tables

**Table 1:** Logistical control requirements [47].

Characteristics	Job Shop Production	Screening for Material Testing
Material flow	Undirected	Undirected
Process chain	Depending on order	Depending on order and aim of analysis
Variety	High	High
Re-routing	Rare case, should be avoided	Common, standard process
Ad hoc changes	Processing according to order content	Schedule changes depending on measured values
